# Intramuscular injection of human chorionic gonadotropin prior to secretory transformation in patients undergoing frozen-thawed embryo transfer cycles

**DOI:** 10.1186/s12958-020-00606-y

**Published:** 2020-05-25

**Authors:** Ling Deng, Xin Chen, Christophe Blockeel, De-Sheng Ye, Shi-Ling Chen

**Affiliations:** 1 Center for Reproductive Medicine, Department of Gynecology and Obstetrics, Nanfang Hospital, Southern Medical University, Guangzhou, China; 2 Center for Reproductive Medicine, Department of Gynecology and Obstetrics, Shunde Hospital, Southern Medical University, Foshan, China; 3 Center for Reproductive Medicine, Universitair Ziekenhuis Brussel, Laarbeeklaan 101, Brussels, 1090 Belgium

**Keywords:** Human chorionic gonadotropin, Frozen-thawed embryo transfer, Pregnancy outcome

## Abstract

**Background:**

The major difference between a natural cycle and an artificially prepared cycle is the lack of luteinizing hormone (LH) peak in the latter. The LH/hCG receptors were identified to express in human endometrium and evidences of experiments also suggested the beneficial role of hCG in embryo implantation, indicating that the LH peak might be of clinical significance and the activation of LH/hCG receptors in the endometrium could improve embryo implantation. Hence, we postulated that the addition of hCG prior to secretory transformation in an artificial cycle might improve pregnancy outcomes.

**Methods:**

This retrospective cohort study was conducted at a Reproductive Medicine Center between 2016 and 2018. Patients aged ≤43 years at the (index) oocyte retrieval and undergoing artificially prepared frozen-thawed embryo transfer (FET) with at least one good-quality embryo transferred were included. The cycles were divided into two groups: The hCG group (*n* = 337) received an intramuscular injection of 10,000 IU hCG before secretory transformation; the control group (*n* = 364) performed FET without hCG administration. The primary endpoint was live birth delivery rate (LBR), secondary outcomes included implantation rate, clinical pregnancy rate (CPR) and ongoing pregnancy rate (OPR).

**Results:**

The LBR (49.9% vs 39.6%, *P <* 0.01), CPR (61.4% vs 50.5%, *P <* 0.01) and OPR (52.8% vs 43.1%, *P <* 0.05) were statistically significantly higher in the hCG group than the control group. The superiority in LBR after hCG administration remained significant after adjusting for confounding factors (OR 1.613, 95% CI 1.173–2.217; *P <* 0.01). In the subgroup analysis, the improvement in LBR was statistically significant after hCG administration for cleavage-stage embryo transfer cycles (51.2% vs 42.3%, *P <* 0.05), whereas for blastocyst transfer cycles, the improvement in LBR was not (45.7% vs 31.3%, *P > 0.05*).

**Conclusions:**

Intramuscular hCG injection prior to secretory transformation may benefit LBR in patients undergoing artificially prepared FET cycles. But it should be noted that nonsignificant tendency towards higher LBR was observed after hCG administration in patients undergoing blastocyst transfer. So, future prospective randomized controlled studies are required to confirm, especially for blastocyst transfer cycles.

## Introduction

Successful embryo implantation is a pivotal step for maintenance of pregnancy, it requires optimal synchronization between the endometrial development and embryo. Implantation failure is still an intractable experience of clinical practice in assisted reproductive technology (ART). According to the ESHRE PGD Consortium data [1], the implantation rate (IR) was 25% for women performing PGS, which was still very unsatisfactory. Obviously, it provides a clue that the endometrial receptivity and the reciprocity between endometrium and embryos play an essential role in achieving implantation.

Human chorionic gonadotropin (hCG), sharing the same receptor as luteinizing hormone (LH), has been used to induce oocyte final maturation or prompt corpus luteum formation by directly acting on LH/hCG receptors in the ovary, traditionally. However, the LH/hCG receptors were also identified to be expressed and be functionally active in human endometrium, with the maximum level in the early-mid secretory phase which exactly covered the “implantation windows” [2]. Independently of its well-known role in the ovary, hCG may have direct effects on the endometrium by acting on LH/hCG receptor [3, 4].

Licht P*,* et al. [5] demonstrated that hCG administration could produce a significant reduction of intrauterine macrophage colony stimulating factor, but an apparent stimulation of leukemia inhibitory factor and vascular endothelial growth factor (VEGF). The expression of these molecules plays an important part in embryos implantation. Besides VEGF, hCG was showed to amplified the responsiveness of endometrial stromal cells to interleukin 1, which could promote the proliferation and motility of human microvascular endothelial cell [6]. It may indicate that hCG would benefit the initiation of angiogenesis and micro-vascularization for preparing a receptive endometrium. The inhibition of insulin-like growth factor binding protein-1 (IGFBP-1) in endometrial stromal cells is stimulated by hCG, which means an important role of hCG on prolongation of the “implantation windows” [7]. Additionally, hCG was reported to induce cytotrophoblast cell proliferation and invasion [8, 9], regulate the maternal-fetal immunologic tolerance [10–12] and suppress myometrial contractility [13, 14]. Although there is no robust evidence, a growing number of clinical trials demonstrate the possible positive impact of hCG administration before embryo transfer (ET) on pregnancy outcomes [15–19].

For frozen-thawed embryo transfer (FET) cycles, the endometrium can be prepared in a natural or in an artificially prepared cycle. Generally, the endometrium is artificially primed by sequential estradiol (E_2_) and progesterone (P) supplementation in an artificial cycle. The major difference between a natural cycle and an artificially prepared cycle is the lack of LH peak in the latter. Mid-cycle hCG administration might rescue the impaired endometrial maturation in patients with very low LH level as reported [20], indicating that the LH peak before secretory transformation might have an effect on endometrial receptivity and the activation of the LH/hCG receptor in the endometrium could improve embryo implantation [3]. Moreover, endometrial epithelium was showed to secrete hCG after synchronously progesterone-stimulated secretory transformation in natural cycle [21]. Therefore, in an attempt to mimic natural physiology, the purpose of the study was to inject hCG intramuscularly prior to secretory transformation. This retrospective study was performed to evaluate the effect of hCG administration before secretory transformation on IR and pregnancy outcomes of artificially prepared FET cycles.

## Materials and methods

### Study population

This retrospective study was conducted at Nanfang Hospital of Southern Medical University (Guangzhou, China) from January 2016 to February 2018. Women aged between 23 and 43 years at the (index) oocyte retrieval and undergoing artificially prepared FET with at least one good-quality embryo transferred and without using GnRH agonists before steroid administration were included. Patients diagnosed with endometriosis were excluded. The cycles were divided into two groups, hCG group and control group, according to the use of hCG prior to secretory transformation or not, which was determined at the physician’s discretion. The trial was approved by the Medical Ethics Committee of Nanfang Hospital, Southern Medical University. The protocols of ovarian stimulation and the laboratory technologies were unchanged over the course of the study. Women of both study groups underwent ovarian stimulation protocols according to the physicians’ discretion, followed by conventional in vitro fertilization (IVF) and/or intracytoplasmic sperm injection (ICSI). Vitrification of the remaining available embryos was carried out.

For day 3 ET, a good-quality embryo was defined as having seven to eight cells and, ≤ 20% fragmentation and no multinucleation. Blastocyst was graded according to the SART embryo grading system [22], and blastocyst with blastocoel fulfilling the whole blastocyst, a good inner cell mass (score A or B) and a trophectoderm layer (score A or B) was viewed as a good-quality embryo.

### Endometrial preparation

Oral E_2_ valerate (Progynova, Schering) was initiated on day 2–4 of the menstrual cycle, at a dose of 4 mg per day (2 mg twice daily) during 4 days, followed by 6 mg per day (2 mg three times daily) during the next 4 days, and then by 8 mg per day (2 mg four times daily) during the next 4 days. On day 12 of E_2_ supplementation, transvaginal ultrasound was performed to measure the endometrial thickness. Endometrial thickness ≥ 8 mm was deemed as an ideal thickness. In cases of inadequate endometrial thickness (i.e. < 8 mm), higher doses of oral estradiol valerate or addition of vaginal E_2_ tablets (Femoston, Abbott) were administered with a maximum E_2_ of 10 mg per day until endometrial thickness reached 8 mm. If the endometrial thickness remained inadequate after 20 days of E_2_ supplementation, the cycle was canceled. When endometrium reached optimal thickness, a blood test was performed to measure serum E_2_ and P levels (If serum P levels were > 1 ng/mL, the cycle was canceled). The use of hCG before secretory transformation was determined at the physician’s discretion. Intramuscular (IM) injections of 10,000 IU hCG (Chorionic Gonadotropin, Livzon) in the morning and 20 mg P in oil (Progesterone Injection, Xianju) in the afternoon, respectively, were administrated in the hCG group. In the control group, no hCG was administrated. On the next day, in both treatment groups, IM progesterone in oil at 40 mg per day was started for luteal phase support (LPS) combined with E_2_ administration. Cleavage-stage ET was performed 3 or 4 days following 40 mg P administration, while blastocyst ET was conducted on the sixth day of 40 mg P administration. Serum β-hCG levels were checked 10–12 days after FET. If serum β-hCG ≥ 50 mIU/ml, LPS was continued up to 9–10 gestational weeks. A transvaginal ultrasonography was performed at 5–6 weeks of gestational age to confirm intrauterine pregnancy and determinate the number of gestational sacs. In case of negative pregnancy test or pregnancy loss, LPS was discontinued.

### Outcome measures

Live birth delivery rate (LBR), defined by the International Committee for Monitoring Assisted Reproductive Technology (ICMART) as live birth beyond 26 weeks of gestational age per FET cycle [23], was the primary endpoint of the study. Secondary outcomes included IR, clinical pregnancy rates (CPR), ectopic pregnancy rates, ongoing pregnancy rates (OPR), and first trimester miscarriage rates. IR was defined as the proportion of the number of gestational sacs to the number of embryos transferred according to ICMART [23]. Clinical pregnancy was defined as one or more intrauterine gestational sacs observed by ultrasound scan at 5–6 weeks of gestational age [23]. A pregnancy outside the uterine cavity, detected by ultrasound, surgical visualization or histopathology, was viewed as ectopic pregnancy [23]. Ongoing pregnancy was defined as a clinical pregnancy beyond 12 gestational weeks, while first trimester miscarriage rate was defined as the percentage of lost pregnancies before 12 gestational weeks relative to clinical pregnancies.

### Statistical analysis

Statistical analysis was conducted using Statistical Package for Social Sciences (ver. 20.0; SPSS Inc., Chicago, USA). For categorical variables, Chi-square (χ^2^) test or Fisher’s exact test was conducted, where appropriate. For continuous variables, at first, the Kolmogorov–Smirnov test was used to determinate the normal distribution of variables and results were given as mean ± standard deviation (mean ± SD) or median (25th–75th centiles) according to the date distribute normally or not, respectively. Logistic regression analysis was conducted to account for potential confounders that would exert independent effects on LBR and then adjust the confounding factors to evaluate the association between the use of hCG and LBR. The statistically significant *p*-value was set at 0.05.

In our preliminary data, LBR were 51% and 40% in patients performing IM hCG prior to ET in artificially prepared cycles and patients who not received IM hCG, respectively. To achieve a power of 80% to detect an 11% difference in LBR, it was calculated that at least 320 cycles in each group would be needed. Consecutive cycles were recruited until the specified sample size in each group was achieved.

## Results

A total of 1216 FET cycles were conducted during the study period. Of these, 486 cycles did not meet the inclusion criteria (279 cycles performing natural cycle or mild stimulation protocol for endometrial preparation, 104 cycles performing artificially prepared FET cycle with GnRH agonists pretreatment before steroid administration, 92 cycles in which no good-quality embryo was transferred and 11 cycles in which the age of patients at the (index) oocyte retrieval patients was more than 43 years) and 29 cycles were excluded for the patients suffering from endometriosis. Thus, a total of 701 artificially prepared FET cycles were included for analysis, 364 of which were in the control group and 337 of which were in the hCG group.

The two groups had similar basic characteristics and clinical parameters of index IVF/ICSI cycle (Table I). Regarding the included FET cycles, they were also comparable in terms of endometrial thickness, number of embryos transferred and good-quality embryos transferred, duration of E_2_ administration and serum E_2_ levels, but the serum P levels in the control group was significantly higher than in the hCG group (Table 1). The mean value (SD) of serum P levels were 0.5 (0.2) and 0.4 (0.2) ng/mL in the control and hCG group (*P* = 0.001), respectively (Table 1).

**Table 1:** Demographic and clinical data of the index stimulated IVF/ICSI cycle and FET cycle

	Control group (***n*** = 364)	hCG group (***n*** = 337)
**IVF/ICSI cycles:**		
Age at index IVF/ICSI (years)	31.4 ± 4.8	31.7 ± 4.7
Duration of subfertility (years)	4.5 ± 3.2	4.4 ± 3.1
Body mass index (kg/m^2^)	21.4 ± 2.9	21.5 ± 3.1
Primary subfertility	169/364 (46.4%)	141/337 (41.8%)
Number of previous ET cycles	1 (0–1)	0 (0–1)
Primary diagnosis		
Tubal	123/364 (33.8%)	111/337 (32.9%)
Uterine	5/364 (1.4%)	4/337 (1.2%)
Anovulation	8/364 (2.2%)	14/337 (4.2%)
Diminished ovarian reserve	8/364 (2.2%)	7/337 (2.1%)
Mixed factors of female	70/364 (19.2%)	77/337 (22.8%)
Male	36/364 (9.9%)	30/337 (8.9%)
Both husband and wife	109/364 (29.9%)	86/337 (25.5%)
Unexplained	5/364 (1.4%)	8/337 (2.4%)
Number of oocytes retrieved	14.5 ± 7.9	15.6 ± 8.5
Number of mature oocytes	12.5 ± 7.0	13.4 ± 7.3
**FET cycles:**		
Maternal age at FET (years)	32.2 ± 5.2	32.5 ± 4.6
First ET cycle	191/364 (52.5%)	178/337 (52.8%)
Endometrial thickness (mm)	9.8 ± 2.0	9.8 ± 1.6
Duration of estradiol administration (days)	14.2 ± 2.9	14.5 ± 2.9
P levels (ng/mL) ^*^	0.5 ± 0.2	0.4 ± 0.2
E_2_ levels (pg/mL)	253.3 (175.7–400.7)	253.6 (179.5–544.2)
Number of embryos transferred	2 (2–2)	2 (2–2)
Number of good-quality embryos	2 (1–2)	2 (1–2)
Single embryo FET cycles	50/364 (13.7%)	50/337 (14.8%)
Single cleavage-stage embryo	18/272 (6.6%)	18/252 (7.1%)
Single blastocyst	32/83 (38.6%)	32/81 (39.5%)
Stage of embryo transferred		
Cleavage stage	272/364 (74.7%)	252/337 (74.8%)
Blastocyst stage	83/364 (22.8%)	81/337 (24.0%)
Both stage	9/364 (2.5%)	4/337 (1.2%)

Data presented as number (percentage) or median (25th–75th centiles) or mean ± SD; Continuous variables were compared between the two groups with Mann-Whitney U-test or with Student’s t-test according to the data distribute normally or not; Categorical variables are compared with Chi-square (χ2) test or Fisher’s exact test

*P*-value non-significant unless specified

^*^P < 0.01

^#^*P* < 0.05

There were 46.5% (294/632) of embryos successfully implanted in the hCG group, which was statistically significantly higher than in the control group (35.7% (246/690), *P* < 0.001). The CPR and OPR were higher in the hCG group as compared with the control group. First trimester miscarriage occurred in 29 (14.0%) of FET cycles with hCG administration and 27 cycles (14.7%) in the control group, which was comparable between the two groups (odds ratio (OR) 0.947, 95% confidence interval (CI) 0.538–1.669; *P* = 0.852). The LBR in the hCG group was 49.9% (168/337) and 39.6% (144/364) in the control group resulting in a relative difference of 26.0% (OR 1.519, 95% CI 1.126–2.049; *P* = 0.006). (Table 2).

**Table 2:** Outcomes of frozen embryo transfer (FET) cycles

	Control group (***n*** = 364)	hCG group (***n*** = 337)	Odds ratio (95% CI)	***P***-value
Implantation rate^*^	246/690 (35.7%)	294/632 (46.5%)	1.570(1.259–1.958)	< 0.001^a^
Clinical pregnancy rate^*^	184/364 (50.5%)	207/337 (61.4%)	1.558 (1.153–2.104)	0.004
Ectopic pregnancy rate	1/364 (0.3%)	3/337 (0.9%)	3.260 (0.338–31.498)	0.280
Ongoing pregnancy rate^#^	157/364 (43.1%)	178/337 (52.8%)	1.476 (1.096–1.988)	0.010
First trimester miscarriage rate	27/184 (14.7%)	29/207 (14.0%)	0.947 (0.538–1.669)	0.852
Live birth delivery rate^*^	144/364 (39.6%)	168/337 (49.9%)	1.519 (1.126–2.049)	0.006

Implantation rate = the number of intrauterine gestational sacs / the number of embryos transferred * 100%;

First trimester miscarriage rate = the cycles of lost pregnancies before 12 gestational weeks / the cycles of clinical pregnancies. * 100%;

^*^P < 0.01

^#^*P* < 0.05

^a^*P =* 0.00006

Univariate logistic regression showed that age at fresh IVF/ICSI attempt (OR 0.901, 95% CI 0.871–0.931; *P* < 0.001), duration of infertility (OR 0.942, 95% CI 0.897–0.989; *P* = 0.017), BMI (OR 0.948, 95% CI 0.901–0.998; *P* = 0.042), number of embryos transferred (OR 1.958, 95% CI 1.315–2.914; *P* = 0.001), number of good-quality embryos transferred (OR 1.991, 95% CI 1.474–2.689; *P* = 0.000), number of previous ET cycles (OR 0.752, 95% CI 0.651–0.868; *P* = 0.000) and the use of hCG (OR 1.519, 95% CI 1.126–2.049, *P* = 0.006) all affected LBR after FET. In the multivariate logistic regression model, after adjusting the above confounding factors, the use of hCG remained a significant positive factor predictive of live birth (adjusted OR 1.613, 95% CI 1.173–2.217; *P* = 0.003).

Subgroup analysis was performed based on the stage of embryos transferred (Table 3). CPR and OPR were statistically significantly improved in the hCG group for women who received a blastocyst transfer. The LBR was also higher in the hCG group compared to the control group, but the difference lacked statistical significance. Nonsignificant trends towards higher CPR and OPR were observed in the hCG group for patients undergoing cleavage-stage ET, but significantly higher LBR was observed in the hCG group.

**Table 3:** Subgroup analysis

	Control group	hCG group	Odds ratio (95% CI)	***P***-value
Blastocyst	*n* = 83	*n* = 81		
Implantation rate*	45/134(33.6%)	68/130(52.3)	2.169(1.320–3.566)	0.002
Clinical pregnancy rate^*^	37/83 (44.6%)	54/81 (66.7%)	2.486 (1.320–4.683)	0.004
Ongoing pregnancy rate^#^	28/83 (33.7%)	41/81 (50.6%)	2.013 (1.072–3.780)	0.029
Live birth delivery rate	26/83 (31.3%)	37/81 (45.7%)	1.844 (0.975–3.487)	0.059
Cleavage-stage embryo	*n* = 272	*n* = 252		
Implantation rate*	196/534(36.7%)	222/492(45.1%)	1.418(1.104–1.821)	0.006
Clinical pregnancy rate	144/272 (52.9%)	151/252 (59.9%)	1.329 (0.940–1.880)	0.108
Ongoing pregnancy rate	126/272 (46.3%)	135/252 (53.6%)	1.337 (0.948–1.885)	0.097
Live birth delivery rate^#^	115/272 (42.3%)	129/252 (51.2%)	1.432 (1.014–2.021)	0.041

^*^P < 0.01

^#^*P* < 0.05

Note: There were 9 cycles and 4 cycles transferred both cleavage stage embryo and blastocyst in control group and hCG group, respectively, which were not included in subgroup analysis

## Discussion

In this large retrospective study, better clinical outcomes (IRs, pregnancy rates and LBRs) were observed when injecting 10,000 IU hCG prior to secretory transformation as compared with cycles without hCG administration in artificially prepared FET cycles. Even after adjusting for the potential confounding factors, the IM hCG injection prior to secretory transformation was still a significant factor predictive for live birth.

This is the second study to investigate the efficacy of IM injection hCG prior to secretory transformation in artificially prepared FET cycles. In a previous study [20], hCG was injected before secretory transformation in oocyte recipients who underwent artificially prepared FET cycles, with or without GnRHa down-regulation. In cycles with GnRHa pretreatment in ovulating women, hCG injection significantly improved implantation rates. The results of our study are in line with these findings, demonstrating significantly higher IR and pregnancy rates when hCG was administered. However, without the use of a GnRHa in non-ovulating patients, hCG administration did not have any impact on implantation, which differs from our study. This difference might be due to the demographic characteristics, such as the inclusion of advanced maternal aged patients (mean age of 42–43 years) as compared with our patients, mostly young women, aged 32 years on average, with different endocrine profiles. A possible explanation may be that the sustained high LH levels in older patients could down-regulate the uterus LH/hCG receptors [2, 24], so that the LH/hCG receptors in the endometrium are not enough for the additional hCG to bind to exert its effect on implantation. Another limitation of their study is the relatively small sample size in order to prove any differences in CPR; besides, LBR was not evaluated.

Besides the IM hCG injection, a number of studies explored the clinical efficacy of intrauterine hCG infusion prior to ET, with different protocols and contradictory results [15–17, 25, 26]. With regard to intrauterine hCG injection, still a number of questions remain to be answered: a. the ideal infusion volume, enough to affect tiny endometrial surface but not the whole uterus, b. the time interval between the hCG infusion and the moment of ET, in order to achieve its maximum effects on implantation via activating the pathway [27]. After adjusting the above disturbing factors, Navali N*,* et al. [19] found the positive role of intrauterine hCG injection in preparation for successful implantation and pregnancy. Moreover, a recent review concluded the beneficial effect of intrauterine hCG infusion in women undergoing ET [28]. Furthermore, evidences of experiments suggested a beneficial effect on embryo implantation by improving endometrial receptivity and angiogenesis, enhancing trophoblast invasiveness and regulating immunologic balance at maternal-fetal interface through interactions with several molecules and cells as mentioned above. To this extent, our study seems to support the hypothesis that the addition of hCG could improve pregnancy outcomes.

However, there are several aspects of intrauterine hCG injection that would have more or less influences on pregnancy outcomes. Inappropriate uterine cavity operation may induce uterine contractions, which was reported to possibly interfere with embryo implantation [29]. And endometrial injury caused by infusion procedure is non-negligible. Otherwise, it should be noted that the surplus infusion fluid may expel embryo from its primary site and interfere the embryo adhesion to the endometrium [30]. But in our study, IM injection is not only a simple procedure without operational heterogeneity for doctors or nurses and additional stress for patients, but could be performed freely that did not rely on the ET time points. And we also confirmed that IM hCG injection also improves IR and pregnancy outcomes.

In the subgroup analysis of our study, we observed pregnancy rates were improved after hCG administration regardless of cleavage-stage embryo and blastocyte transfer cycles. It should be noted that pregnancy outcomes in blastocyte transfer cycles were inferior to those in cleavage-stage ET cycles. This mainly be due to most of blastocyte transfer cycles (39.0%, 64/164) performing single ET in our center whereas a small fraction of cleavage-stage ET cycles (6.9%, 36/524) conducting single ET policy, considering that the significantly higher LBR was observed in blastocyte transfer cycles than in cleavage-stage ET cycles [31].

For blastocyte transfer cycles, the improvement in CPR and OPR were statistically significant after hCG administration whereas the improvement in LBR was not. The relative increase in LBR following hCG injection was 46.0% as compared to control group, so the lack of statistical significance may relate to the insufficient sample size. Otherwise, it also should be considered that the observed superiority might be decreased after more participants been recruited. As for cleavage-stage ET cycles, the statistically significant improvement only was found in LBR after hCG administration, the increase in CPR and OPR was also observed between groups but lack of statistical significance. Furthermore, the relative increase in LBR following hCG injection was also higher in blastocyte transfer cycles (46.0%) than in cleavage-stage ET cycles (21.0%), despite the sample size was not large enough, which indicated that the hCG addition appeared to exert a better effect on pregnancy outcomes of blastocyst transfer cycles as compared with cleavage-stage ET cycles.

HCG starts expressing at cleavage-stage embryos and is increasingly produced by cytotrophoblast cells after implantation, indicating the first 2 days of endogenous hCG production from cleavage-stage embryo onward may be of clinical importance [32, 33]. The hCG addition in our study might exactly make up the lack of endogenous hCG effect in endometrium during the interval from cleavage-stage to blastocyte since the long half-time period of hCG. Otherwise, only considering the beneficial effect of hCG on endometrium suggested by in vitro and in vivo evidences, there existed another possibility that the initial time and dose of hCG addition in the present study may be not optimal for cleavage-stage ET cycles, since the interval time between hCG administration and cleavage-stage ET is too short to produce the beneficial effects of hCG administration on endometrium promptly and sufficiently. Whether a higher hCG dose, an earlier hCG injection or an additional hCG injection could preferably improve pregnancy outcomes in cleavage-stage ET cycles should be further investigated.

Empirically, the ideal dose of hCG to induce oocyte maturation is 4000–10,000 IU in IVF/ICSI cycles, which could produce a satisfactory fertilization rate [34]. To date there were no studies reporting that a higher dose of hCG could impair embryo implantation, several trials found pregnancy rates to be comparable among the varying doses of hCG [34, 35] and even a significantly higher CPR in the higher dose of hCG group was observed [36]. A higher dose of hCG not only influences the amplitude of serum hCG levels, but also the duration in which hCG levels are maintained above the threshold [34]. To give enough time and levels for hCG to achieve its effect on embryo implantation, we injected 10,000 IU hCG. Although the use of hCG (10,000 IU) in our study was proved to improve the pregnancy outcomes, the optimal dose and time point of hCG injection needs to be determined in further studies, especially in cleavage-stage ET cycles.

Strength of this study was its large sample size. However, due to its retrospective design, findings may be limited by the underlying selection bias and confounding factors that have correlation with pregnancy outcomes, such as female age, number of embryos transferred and number of good-quality embryos transferred. But the difference in LBR remained statistically significant even after adjusting these small differences among the above factors by multivariable regression analysis. Furthermore, it is possible that not all confounding factors have been counted in this retrospective study, such as patients’ psychological effects. Another problem is that the serum P levels were statistically significantly different between the control and hCG group. In our center, if the serum P levels were > 1 ng/ml, the FET cycles would be canceled and the mean value (SD) of serum P levels in the present study were 0.5 (0.2) and 0.4 (0.2) ng/mL in the control and hCG group, respectively, which were less than the levels that could impair the pregnancy outcomes [37].

In summary, this study suggested that IM hCG injection prior to secretory transformation may have a beneficial effect on the clinical outcomes of artificially prepared FET cycles. The tendency towards higher pregnancy rates with the IM hCG injection certainly encourages us to conduct a lager RCT in order to test the clinical efficacy prior to apply i.m. hCG into clinical practice. The slightly different clinical efficacy of IM hCG injection prior to secretory transformation between cleavage-stage embryo and blastocyst transfer cycles, also suggests that different starting time of hCG injection, different dosage regimens also need to be investigated by further randomized studies, especially for blastocyst.

## Data Availability

All data generated or analyzed in the present study are included in this published article.

## Abbreviations

ART: Assisted reproductive technology
IR: Implantation rate
hCG: Human chorionic gonadotropin
LH: Luteinizing hormone
VEGF: Vascular endothelial growth factor
IGFBP-1: Insulin-like growth factor binding protein-1
ET: Embryo transfer
FET: Frozen-thawed embryo transfer
IVF: In vitro fertilization
ICSI: Intracytoplasmic sperm injection
E_2_: Estradiol
P: Progesterone
IM: Intramuscular
LPS: Luteal phase support
LBR: Live birth delivery rate
CPR: Clinical pregnancy rates
OPR: Ongoing pregnancy rates
OR: Odds ratio
CI: Confidence interval
